# Cholera at Salonica

**Published:** 1921-06-18

**Authors:** 


### CHOLERA AT SALONICA.

BY A SOCIAL WORKER.

Salonica is for us a place of associations that have no connection with a native home life. "There is always trouble in the Balkans," and we cannot wonder that one of the first writers of local sketches and stories, M. Albert Asséo, bears a heavy heart which tips his pen with melancholy. Yet there is a fine power of description and a tender love of home and country in his book "Das Masseng'zab," which unfortunately will not be available to many English readers at present, as it has been written in German and published in Switzerland.

One of his studies deals with the experience of his own family during the cholera epidemic of 1913 at Salonica, and we feel ourselves almost taken back to the Middle Ages as we read his Defoe-like account of the brooding horror of the streets, the odour of quicklime, the silence broken at times by the rumbling of heavy, closed vans driven by cloaked and masked figures, or by sudden outcries and lamentations from the railed-in houses, where soldiers stood ready to close with their fixed bayonets any door suddenly opened. Foot-passengers followed zig-zag paths in the street to avoid contact with others, though, if friend forgetfully touched friend, each would take a little bottle from his pocket and disinfect the erring hand.

In the street where the Asséo family lived only three houses were in a short time left untouched. Each morning the one son rose early, took up the post of watch-dog at the house door, and only varied his morning task of keeping others from entering by expeditions upstairs to keep his mother and sisters from looking out of the closely shut windows.

The day came when the house opposite was at last stricken, and a terrible scene took place, the muffled removers of the dead tearing a young husband from the bride who had been brilliantly well a few hours before, and a widow from the corpse of her only son. At evening husband and mother were fetched in their turn. Then the window of another apartment in the same house was flung wide, and the wife of the hardworking father and breadwinner of a large family cried to her neighbours to ask their prayers—her husband lay in the cholera-agony. From many windows other mothers answered her, but Madame Asséo did more. "I am coming across to you," she called, unable to let her friend suffer alone. Husband and daughters gathered round her and entreated, but she tried to go till her one son, Albert, looked speechlessly in her face, admiration for her self-forgetfulness striving with terror. . . . Then suddenly her resistance collapsed. "Did you think I could leave you?" she murmured, and turned back to her family. But the sick man recovered: he was nearly, or quite, the first to do so of those who had sickened.

It was well that the brave woman had not gone. Her son went out for necessary shopping, came home, and sat down to supper. Then suddenly the hideous thing had him in its grip, and, despite all efforts to seem well, the pain and fever betrayed the case. The mother saw, and lied bravely.

"The doctor says we are immune." But she gave the medicine that was kept in readiness, ordered water for a hot bath, rubbed her son's body with hot oil, bandaged the abdomen closely, laid him in bed, and then sat down beside him and demanded that the member of the family who could tell the best story should do so at once, for that she had a mind to be amused!

It was Albert who responded. While his body fought as with a raging beast, he told the story of Mary Stuart at full length and with the interest of one whose imagination the romance had lately gripped.

It was a tremendous effort, but deep called to deep; the son's courage rising to meet the mother's intensity of saving purpose. He talked on till it began to seem that the wild beast that struggled within him to choke and kill was beginning to tire. The attacks of pain grew less violent; there were longer intervals between them: they almost ceased.

Then suddenly his head seemed heavy as lead, his eyelids dropped, an intolerable desire to sleep came over him. He lay back and gave way to it. When he woke, refreshed and well, it was broad day. At the foot of his bed sat one of the sisters, a piece of needlework in her hands. By the bedside his mother was standing: he had an impression that she had stood so all night.

